# The Effectiveness of a Single Hyaluronic Acid Injection in Improving Symptoms and Muscular Strength in Patients with Knee Osteoarthritis: A Multicenter, Retrospective Study

**DOI:** 10.3390/jpm14080784

**Published:** 2024-07-24

**Authors:** Domiziano Tarantino, Alfonso Maria Forte, Antonio Picone, Felice Sirico, Carlo Ruosi

**Affiliations:** 1 Department of Public Health, University of Naples Federico II, 80131 Naples, Italy; antonio.picone@unina.it (A.P.); sirico.felice2@unina.it (F.S.); caruosi@unina.it (C.R.); 2 Biomedical Research Center, Gruppo Forte, 84124 Salerno, Italy; a.forte@gruppoforte.it

**Keywords:** osteoarthritis, knee osteoarthritis, hyaluronic acid, injection, isokinetic dynamometer

## Abstract

Knee osteoarthritis (KOA) is a progressive and multifactorial disease that leads to joint pain, muscle weakness, physical disability, and decreased quality of life. In KOA, the quantity of hyaluronic acid (HA) and the molecular weight (MW) are decreased, leading to joint pain due to increased wear of the knee articular cartilage. Arthrogenic muscle inhibition, which is usually found in patients with KOA, is associated with joint inflammation, pain, and swelling, also causing muscle atrophy, primarily of the anterior thigh muscles, and hindering the rehabilitation process. The aim of our work was to determine if a single HA infiltration could minimize the effects of arthrogenic muscle inhibition in patients with KOA in the short term, using isokinetic dynamometry to evaluate the strength of the knee extensor and flexor muscles of the thigh. Thirty patients with KOA who underwent both clinical and isokinetic assessment, and that received a single injection of HA, were retrospectively included. Our results showed that a single intra-articular injection of HA significantly reduces pain and improves joint function at four weeks, while non-statistically significant improvements were observed for the reference isokinetic parameter (maximum torque) at both 90°/s and 180°/s. Further high-quality studies are necessary to confirm the results of our study.

## 1. Introduction

Osteoarthritis (OA) is a chronic and degenerative disease affecting the joints, with the knee being the most frequently affected site (about 50% of all OA cases) [1,2,3,4]. 

KOA is a progressive and multifactorial disease that leads to joint pain, muscle weakness, physical disability, and decreased quality of life. The pathogenic process of OA is characterized by mechanical, inflammatory, and metabolic factors leading to an imbalance between the repair and destruction of joint structures [5,6]. Risk factors for KOA include age, genetics, body weight, sex, ethnicity, and previous injuries (work/sport activities) [7,8,9,10]. 

During KOA development, the balance between the synthesis and degradation of articular cartilage is mainly influenced by pro-degenerative cytokines such as matrix metalloproteinases (MMPs), interleukins (IL) (e.g., IL-1β, -6, -15), tumor necrosis factor-α (TNF-α), and reactive oxygen species (ROS). These factors degrade the extracellular cartilage matrix (ECM), inhibit the proliferation of articular chondrocytes, and promote their apoptosis, causing an imbalance in the synthesis and degradation of collagen and proteoglycans (PGs) in the cartilage matrix, leading to a loss of cartilage elasticity, deterioration, and breakdown, eventually resulting in the erosion of the joint surface [11,12].

KOA is mainly diagnosed based on signs and symptoms (pain, functional limitation, hypertrophy, and swelling). According to the new National Institute for Health and Care Excellence (NICE) guidelines, KOA can be diagnosed clinically without imaging in patients aged 45 and older who present with activity-related joint pain [13]. Imaging tests (such as weight-bearing radiographs or magnetic resonance imaging) are indicated only when the presentation is atypical (e.g., prolonged joint stiffness, rest pain) or when there is an unexpected rapid progression of symptoms or a change in clinical characteristics (e.g., swollen and hyperemic knee with nocturnal pain) [14].

The first-line treatment for KOA is conservative and includes non-pharmacological approaches such as physical exercise (strength, aerobic and aquatic, and stretching and proprioceptive exercises) [15,16,17,18,19], physiotherapy and physical therapies (transcutaneous electrical nerve stimulation (TENS), diathermy, etc.) [20,21,22,23], weight loss (if obese or overweight), use of walking aids or braces (if indicated), education, and self-management (maintaining good lifestyle habits, regular exercise, weight control, and avoiding heavy weight-bearing activities), and pharmacological approaches such as paracetamol, non-steroidal anti-inflammatory drugs (NSAIDs) [1,24], chondroprotective agents (glucosamine sulfate and chondroitin sulfate) [25,26,27], and intra-articular therapies using corticosteroids (CS) [15], hyaluronic acid (HA), and platelet-rich plasma (PRP) [28,29,30,31,32]. 

HA is one of the main components of synovial fluid and plays several key roles in maintaining cartilage health and intra-articular homeostasis [33]. Its rheological and viscoelastic properties provide significant lubricating and shock-absorbing capabilities to the synovial fluid, while its macromolecular size and hydrophilicity help retain fluid in the joint cavity during movements and even after the removal of forces due to mechanical loading [34].

Additionally, HA interacts with pro-inflammatory mediators and, by binding to its cellular receptors, modulates cell proliferation, migration, and gene expression within the joint. The progression of KOA results from a reduction in the molecular weight (MW) (from 6500–10,900 kDa to 2700–4500 kDa) [35] and the concentration of HA in the synovial fluid [34]. This occurs due to hyaluronidases (enzymes that degrade HA) [36,37], ROS, and nitric oxide synthase (NOS) [38]. This degradation of synovial fluid causes joint pain due to increased wear of the knee articular cartilage [35].

Due to the direct association between HA degradation and KOA progression, the intra-articular administration of exogenous HA represents a well-established therapeutic option [39]. Intra-articular HA treatment aims to fill the joint space to counteract the reduction in HA concentration and distribution that occurs with KOA progression [35]. The binding of HA to its CD44 receptor leads to numerous beneficial effects such as chondroprotection, anti-inflammatory effects (thanks to the reduction of IL-1β expression and subsequent reduction of MMPs release), and an increased synthesis of PG and glycosaminoglycans (GAGs) [35].

Joint pain and the consequent alteration of daily activities often lead to an overall reduction in motor activity levels. This can result in the weakening of the extensor and flexor muscles of the thigh, with a secondary increase in joint instability and degeneration [40]. Specifically, in KOA, there is up to a 50% reduction in quadricep strength [41,42]. This occurs partly due to a neural activation deficit in the quadriceps known as arthrogenic muscle inhibition associated with joint inflammation, pain, and swelling [43,44]. Arthrogenic muscle inhibition causes muscle atrophy, primarily of the anterior thigh muscles, and hinders the rehabilitation process [33,44,45,46]. 

There are various options for assessing lower limb muscle strength in the presence of KOA, including manual muscle testing, handheld dynamometry, and isokinetic dynamometry [47]. A recent literature review has demonstrated that isokinetic testing is a useful and valid tool for assessing muscle strength in patients with KOA [47].

In our study, we hypothesized that a single HA infiltration could minimize the effects of arthrogenic muscle inhibition in patients with KOA in the short term, using isokinetic dynamometry to evaluate the strength of the knee extensor and flexor muscles. Given that scientifically validated questionnaires and rating scales are primarily based on patients’ subjective perceptions, our goal was to make them more objective through the use of isokinetic methodology.

## 2. Materials and Methods

All records containing clinical and radiological information about patients admitted with KOA to the Rehabilitation and Spinal Deformities Unit of the University of Naples Federico II and the outpatient clinics of the Gruppo Forte in Salerno between May and November 2023 were retrieved from the centers’ databases.

### 2.1. Inclusion and Exclusion Criteria

The inclusion criteria were as follows: age between 40 and 70 years and diagnosis of primary KOA according to the American College of Rheumatology (ACR) guidelines [48].

The exclusion criteria were as follows: inflammatory diseases (e.g., rheumatoid arthritis, etc.), joint infections, acute synovitis, neuropathic arthropathy (Charcot), gout, intra-articular tumors, varus or valgus deformities >15°, ligamentous instability, previous major traumas (such as ligament injuries or fractures) and knee surgeries, avascular necrosis, Paget’s disease, major dysplasia or congenital anomalies, osteonecrosis, acromegaly, hemochromatosis, Wilson’s disease, primary osteochondromatosis, Ehlers–Danlos syndrome, hyperparathyroidism, and hypothyroidism. Additionally, having received intra-articular injections in the knee in the six months prior to the study was also an exclusion criterion.

### 2.2. Clinical and Functional Evaluation

The patients included in the study received, at the time of assessment, a clinical evaluation, followed by an isokinetic strength test, and finally an HA injection.

Age, sex, weight (in kilograms), height (in meters), BMI (kg/m^2^), employment status (or former employment if retired), physical activity, comorbidities and medications taken, dominant limb, imaging exams performed, knee(s) affected by KOA, and the severity of KOA were retrieved.

Radiological evidence of KOA was assessed using the Kellgren–Lawrence (K-L) classification for radiographs [49] and a classification highly correlated with K-L grades for MRIs [50]. 

Pain intensity was assessed using the Visual Analogue Scale (VAS) [51], while functional assessment was performed using the Knee injury and Osteoarthritis Outcome Score (KOOS) in its Italian version [52]. Both assessments were conducted at T0 (the day of the first isokinetic test and injection) and at T1 (four weeks after T0).

### 2.3. Isokinetic Assessment

The isokinetic evaluation of the strength of the extensor and flexor muscles was performed using the Easytech “Prima Doc”^®^ (Easytech, Borgo San Lorenzo, Italy) isokinetic dynamometer. 

**Figure 1:**
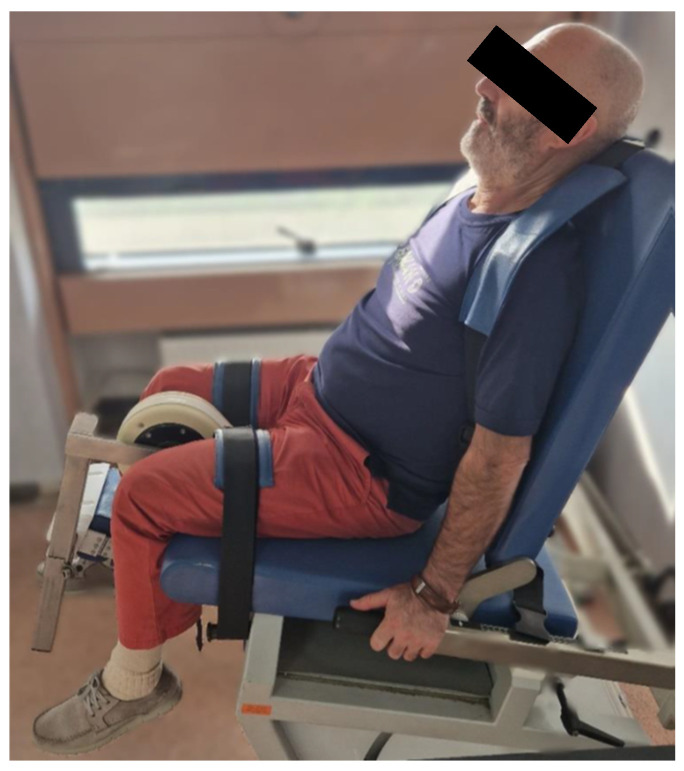
Patient’s position on the isokinetic dynamometer.

Subjects, after a brief theoretical explanation regarding the isokinetic test, were seated on the machine’s chair with their torso and legs well extended, and the inclination (approximately 110°) and depth of the backrest were adjusted to ensure maximum comfort during the test. The straps at the backrest and along each thigh were applied to help maintain stability and overall safety during the examination (Figure 1).

**Figure 2:**
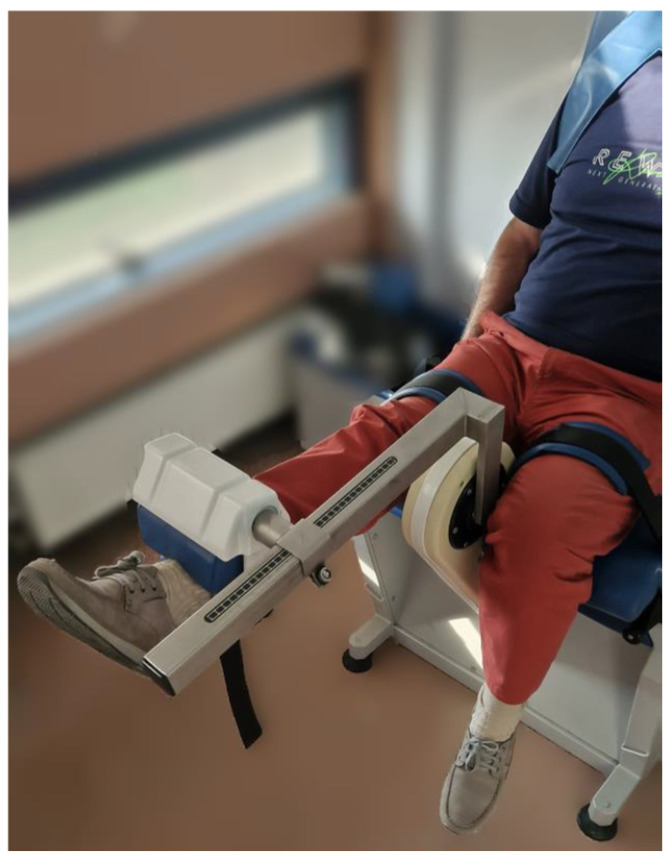
Positioning of the limb to be tested for isokinetic evaluation at the 0 point (maximum extension).

The evaluation of quadriceps strength was, therefore, carried out in a seated position with the hip flexed and the leg flexed at 90° on the thigh, with the dynamometer probe placed three centimeters from the medial malleolus. The ROM used for the test was 0°–90° and was calculated by taking, as a reference for the ninetieth degree, the position of maximum active extension of each individual subject (then, by flexing the leg at 90° on the knee, the 0 point was obtained, i.e., the starting point of each repetition of the test) (Figure 2).

Before starting the test, gravity compensation was performed according to the machine’s software instructions. The protocol, shared and implemented at both centers where the study was conducted, included a warm-up phase followed by a testing phase, which comprised both a strength test (five maximal repetitions—5 RM) and an endurance test (20 maximal repetitions—20 RM).

The warm-up consisted of two sets of 15 repetitions, with the first set “empty” and the second set performed at an angular velocity of 180°/s, and the examinee was advised to apply sub-maximal contractions to progressively get used to the isokinetic muscle effort.

Five minutes after the warm-up phase, the actual isokinetic test began, which included the strength test (5 RM) at an angular velocity of 90°/s and, after a 30-s pause, the endurance test (20 RM) at an angular velocity of 180°/s.

Each patient was encouraged in a standardized manner to exert maximum voluntary effort throughout the execution of the tests. If the same patient was a candidate for isokinetic evaluation and HA infiltration in both knees, a five-minute rest was observed between the two isokinetic tests. The order of test execution (e.g., right knee first and then left) at T0 was recorded to repeat it in the same manner at T1.

Among the many parameters that can be analyzed, for this study, we chose to consider the maximum torque (Max MDF) for both the strength and endurance tests. Each test was recorded by the isokinetic software with the relevant graph showing the trend of Max MDF for individual repetitions in relation to the degree of extension. 

### 2.4. Injection Treatment

**Figure 3:**
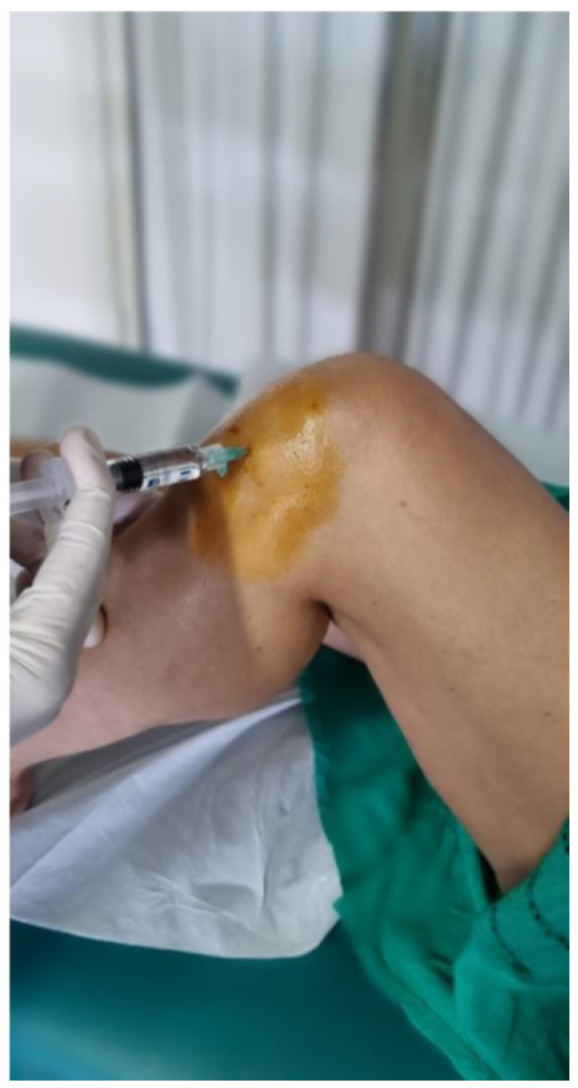
Injection procedure through the inferior-patellar lateral portal.

Patients were treated with a single injection of 60 mg of medium MW HA (1500–2000 kDa)/4 mL (Hyalubrix^®^ 60, Fidia Farmaceutici S.p.A., Abano Terme, Italy) [53,54]. The injection was performed approximately 15 min after the isokinetic test ended. The injection was performed without ultrasound guidance, with the patient in a supine position and the knee flexed, using a 21 G needle (0.8 mm × 40 mm) through the inferior-patellar lateral portal (Figure 3) after appropriate skin disinfection with chlorhexidine and povidone–iodine.

### 2.5. Post-Intervention Recommendations

In the case of post-injection pain, patients were advised to apply ice two to three times a day in the days following the injection and, only in cases of severe pain, to take NSAIDs or pain relievers for no more than three days. 

Patients were also recommended not to undergo any physiotherapy and/or therapeutic exercise and to avoid taking NSAIDs or pain relievers (except as mentioned above) between T0 and T1 to avoid affecting the study results.

### 2.6. Statistical Analysis

Data were analyzed using SPSS statistical software (v.29). Results are presented as mean values and standard deviation (SD). Comparisons for VAS, KOOS, and Max MDF values between T0 and T1 were performed using the paired *t*-test. A *p*-value < 0.05 was considered statistically significant. 

It was not possible to compare knees with different degrees of radiological severity due to the disparity in sample sizes, as the majority of the evaluated knees (34 out of 46, 74%) had a K-L grade II, with only two knees of grade I and 10 of grade III.

## 3. Results

Thirty patients and a total of forty-six knees were included in this study, with two knees being classified as K-L I, thirty-four as K-L II, and ten as K-L III. The anthropometric characteristics of the included patients are reported in Table 1 and Table 2.

What can be inferred from Table 1 and Table 2 is that the sample consisted predominantly of women (16 out of 30) who had a higher average age and BMI compared to men, confirming the higher prevalence of KOA in females [55,56,57]. Age and BMI were directly related to higher K-L scores, confirming the important role of these factors in the pathogenesis and severity of KOA [58,59]. 

Sixteen patients exhibited bilateral knee osteoarthritis (six men, ten women), and in ten of these patients with bilateral osteoarthritis but different K-L grades, the dominant limb was the one with the higher grade.

The changes in VAS between T0 and T1 are reported in Table 3, while the changes in KOOS between T0 and T1 are reported in Table 4.

What can be inferred from Table 3 and Table 4 is that both VAS and KOOS improved significantly between T0 and T1 in all K-L groups, both when patients were evaluated as a whole and within various radiological severity subgroups according to K-L.

Table 5 and Table 6 report the changes in the strength of extensor and flexor muscles between T0 and T1 for each test performed.

What can be inferred from Table 5 and Table 6 is that, concerning Max MDF, the changes between T0 and T1 were not statistically significant, despite a trend towards improvement (both for Max MDF at 5 RM and 20 RM) considering all K-L groups.

## 4. Discussion

In patients with KOA, the reduction in muscle strength, known as arthrogenic muscle inhibition, is associated with inflammation, pain, and joint swelling, resulting in reduced joint mobility and muscle atrophy [43,44].

In our study, we hypothesized that a single HA injection could minimize the effects of quadriceps muscle arthrogenic inhibition and help improve conservative treatment of KOA. We designed a retrospective study to test the efficacy of intra-articular HA for KOA using the VAS and KOOS scales for pain and joint function, as well as the isokinetic test to evaluate the strength of knee extensor and flexor muscles.

Our results showed that a single intra-articular HA injection significantly reduced pain and improved joint function at four weeks. These results are comparable to those obtained in other studies using the same type of HA (Hyalubrix^®^) for KOA [54,60,61,62,63,64,65], even though they followed a protocol of three injections (30 mg/2 mL, one injection per week for three consecutive weeks) instead of a single one as in our study.

The reduction in pain and improvement in joint function could be attributed to the restoration of viscoelasticity and maintenance of knee joint lubrication [66]. Additionally, HA improves chondrocyte survival and stimulates the production of PGs and endogenous HA by suppressing pro-inflammatory factors [67]. The overall result of these effects from intra-articular HA administration is the inhibition of joint inflammation and synovial hypertrophy. 

HA also reduces pain through a direct effect on nerve afferents by modulating the sensitivity of nociceptor nerves [67,68]. Furthermore, in some studies, HA has improved proprioception and muscle strength in the isokinetic test in KOA patients [69].

In our study, non-statistically significant improvements were observed for Max MDF at both 90°/s and 180°/s. The exact reason for the (albeit non-statistically significant) improvement in muscle strength in the isokinetic test after intra-articular HA injection is unknown. This could be due to the reduction in friction related to the increased viscosity induced by HA, leading to reduced joint inflammation and pain, and improved joint function and muscle activation [40,69,70].

Only six studies in the literature have evaluated the efficacy of intra-articular HA administration on muscle strength in KOA patients using an isokinetic dynamometer [33,40,69,70,71,72], and a direct comparison of our results with published studies is difficult mainly due to different isokinetic protocols, HA administration protocols, and types of HA used. Additionally, in some studies, the efficacy of HA was not evaluated alone but combined with other therapies, making it impossible to discern the effects of each individual treatment in the combined therapeutic protocol. 

In four studies [40,69,70,72], the efficacy of HA alone was evaluated either alone or compared to control groups, while in two studies [33,71], the efficacy of intra-articular HA administration was not evaluated alone but in combination with other treatments. 

In these six studies in the literature, pain and functional limitation improved significantly, as also reported in the results of our study. Muscle strength evaluated by isokinetic testing improved in all parameters in three studies [33,40,70], while in the study by Tang et al. [72], strength improved globally at 80°/s but not at 240°/s for the extensor muscles, and in the study by Diracoglu et al. [69], strength improved at 60°/s but not at 180°/s and 240°/s. In the study by Bayramoğlu et al. [71], however, strength did not improve significantly at either 60°/s or 90°/s. 

Comparing our results with those in the literature, the only study [71] that included an isokinetic test at 90°/s (5 RM, as in our study) did not report significant improvements at short-term follow-up (three weeks, whereas our study’s follow-up was four weeks) using both medium and high MW HA. This result is nearly identical to that in our study, although the exact degree of clinical and radiological severity of patients in the referenced study is unknown.

Three studies [33,40,69] included an isokinetic test at 180°/s, with significant strength improvements in two studies at six [40] and eight [33] weeks using the same injection protocol (five injections, one per week) and the same low MW HA. In one study, patients had K-L grades I and II [40], while in the other, they had moderate severity according to Altman [33]. One study [69] did not report significant strength improvements in the isokinetic test at 180°/s at four weeks (as in our study) using three weekly high MW HA injections in patients with K-L II and III. This latter result is nearly identical to that of our study, despite using a different HA and injection protocol.

What can be concluded from the literature is the inconsistency of results regarding changes in muscle strength in isokinetic testing. Indeed, even in studies that evaluated Max MDF at the same angular velocities, using the same type of HA and the same injection protocol, the results were contrasting [69,70,71].

### Strengths and Limitations

The main strength of our study was that a single HA injection was sufficient to achieve a significant improvement in clinical and functional outcomes in the short term. This finding confirms the efficacy reported in other studies [73,74] and is noteworthy given the lack of consensus in the scientific literature regarding the most effective injection protocol (single dose or multiple doses) for the treatment of KOA [75,76,77].

One of the limitations of our study is the small sample size and the lack of a control group. However, we specifically aimed to assess whether the lubricating and anti-inflammatory properties of HA can counteract the arthrogenic muscle inhibition typically observed in patients with KOA. Therefore, a comparison with a control group using other drugs or interventions (e.g., saline injections or physiotherapy) was beyond the scope of this study.

A statistically significant improvement in muscle strength may not have been observed due to the short-term follow-up (four weeks), considering that most studies in the literature have a minimum follow-up of six weeks [33,40,70,72]. A reassessment of patients after three months might have revealed a statistically significant improvement in muscle strength, while a larger sample size might have allowed us to provide more insights into the indirect effects of HA on muscle strength.

## 5. Conclusions

Our results showed that a single intra-articular injection of HA significantly reduces pain and improves joint function at four weeks, while non-statistically significant improvements were observed for Max MDF at both 90°/s and 180°/s. Further high-quality studies (such as randomized clinical trials) evaluating the effects of HA administration on muscle strength in comparison with other therapies, with a larger number of patients and longer follow-up periods, are necessary to confirm the results of our study.

## Figures and Tables

**Table 1:** Anthropometric data. The values are presented as mean and standard deviation (in parentheses).

	Total Sample (*n* = 30)	Males (*n* = 14)	Females (*n* = 16)
Age	58.35 (8.82)	52.57 (8.6)	62.63 (5)
BMI (kg/m^2^)	26.42 (2.71)	25.34 (2.11)	27.37 (2.95)
Knees (DX:SX)	26:20	12:8	14:12
Active workers	22/30	14/14	8/16
Physical exercise	14/30	6/14	8/16

**Table 2:** Anthropometric data and correlation with K-L severity grades. The values are presented as mean and standard deviation (in parentheses).

	K-L I	K-L II	K-L III
Knees (DX:SX)	0:2	16:18	10:0
Age	40	58.29 (8.64)	62.2 (5.12)
Sex (M:F)	2:0	12:16	4:6
BMI (kg/m^2^)	26.47	26.65 (2.61)	27.38 (3.14)
Active workers	2/2	20/28	8/10
Physical exercise	0/2	14/28	4/10

**Table 3:** Changes in VAS between T0 and T1. The values are reported as mean and standard deviation (in parentheses). An asterisk indicates the presence of a statistically significant difference.

	T0	T1	*p*-Value
VAS (all K-L groups)	4.7 (2.1)	1.6 (1.34) *	<0.001
VAS (K-L I)	2	0	/
VAS (K-L II)	4.6 (1.9)	1.6 (1.37) *	<0.001
VAS (K-L III)	5.8 (2)	1.8 (1.3) *	<0.001

**Table 4:** Changes in KOOS between T0 and T1. The values are reported as mean and standard deviation (in parentheses). An asterisk indicates the presence of a statistically significant difference.

	T0	T1	*p*-Value
KOOS (all K-L groups)	75% (0.1)	91% (0.1) *	<0.001
KOOS (K-L I)	96%	100%	/
KOOS (K-L II)	76% (0.1)	91% (0.1) *	<0.001
KOOS (K-L III)	70% (0.1)	91% (0.1) *	<0.001

**Table 5:** Changes in Max MDF of extensor (EXT) and flexor (FLX) muscles at 5 RM (90°/s). The values are reported as mean and standard deviation (in parentheses).

	T0	T1	*p*-Values
EXT:FLX (N/m) (all K-L groups)	88.7(22.8):50.1 (18.5)	92.3 (31.7):51 (22.5)	0.15:0.70
EXT:FLX (N/m) (K-L I)	142:101	171:119	/
EXT:FLX (N/m) (K-L II)	88.8 (21.7):48.7 (15.7)	89.6 (27.6):49.2 (18.7)	0.68:0.68
EXT:FLX (N/m) (K-L III)	77.4 (10.8):44.6 (14.2)	85.8 (29):43.2 (11.3)	0.34:0.63

**Table 6:** Changes in Max MDF of extensor (EXT) and flexor (FLX) muscles at 20 RM (180°/s). The values are reported as mean and standard deviation (in parentheses).

	T0	T1	*p*-Values
EXT:FLX (N/m) (all K-L groups)	68.3 (17.7):42.5 (16)	69.7 (18.7):41.7 (12.7)	0.45:0.61
EXT:FLX (N/m) (K-L I)	77:37	90:44	/
EXT:FLX (N/m) (K-L II)	69 (18.2):43.5 (15.8)	68.4 (17.5):42.5 (13.3)	0.71:0.66
EXT:FLX (N/m) (K-L III)	64.2 (17.2):40 (18)	70.2 (24.3):38.4 (12.7)	0.38:0.64

## Data Availability

The data are not publicly available due to data protections regulations.
